# Influence of pressure on the transport, magnetic, and structural properties of superconducting Cr_0.0009_NbSe_2_ single crystal

**DOI:** 10.1039/c9ra09603e

**Published:** 2020-04-01

**Authors:** K. Manikandan, Rukshana Pervin, C. Saravanan, M. Sathiskumar, Nirman Chakraborty, Parasharam M. Shirage, Swastik Mondal, Velaga Srihari, Himanshu Kumar Poswal, S. Arumugam

**Affiliations:** Centre for High Pressure Research, School of Physics, Bharathidasan University Tiruchirappalli-620024 India sarumugam1963@yahoo.com +91 431 2407045 +91 431 2407118 +91 9500910310; Discipline of Metallurgy Engineering and Materials Science & Physics, Indian Institute of Technology Indore Simrol Campus, Khandwa Road Indore 453552 India; CSIR-Central Glass and Ceramic Research Institute Jadavpur Kolkata 700 032 India; High Pressure and Synchrotron Radiation Physics Division, Bhabha Atomic Research Centre Mumbai 400085 India

## Abstract

We investigate the superconducting critical current density (*J*_c_), transition temperature (*T*_c_), and flux pinning properties under hydrostatic pressure (*P*) for Cr_0.0009_NbSe_2_ single crystal. The application of *P* enhances *T*_c_ in both electrical resistivity (∼0.38 K GPa^−1^: 0 ≤ *P* ≤ 2.5 GPa) and magnetization (∼0.98 K GPa^−1^: 0 ≤ *P* ≤ 1 GPa) measurements, which leads to a monotonic increase in *J*_c_ and flux pinning properties. The field-dependent *J*_c_ at various temperatures under *P* is analyzed within the collecting pinning theory and it shows that *δT*_c_ pinning is the crossover to *δl* pinning above the critical pressure (*P*_c_ ∼0.3 GPa). Our systematic analysis of the flux pinning mechanism indicates that both the density of pinning centers and pinning forces greatly increase with the application of *P*, which leads to an enhancement in the vortex state. Structural studies using synchrotron X-ray diffraction under pressure illustrate a stable hexagonal phase without any significant impurity phase and lattice parameter reduction with *P* shows highly anisotropic nature.

## Abstract 2

We investigate the superconducting and vortex dynamics properties on Cr intercalated NbSe_2_ single crystal by the application of external pressure.

## Graphical abstract

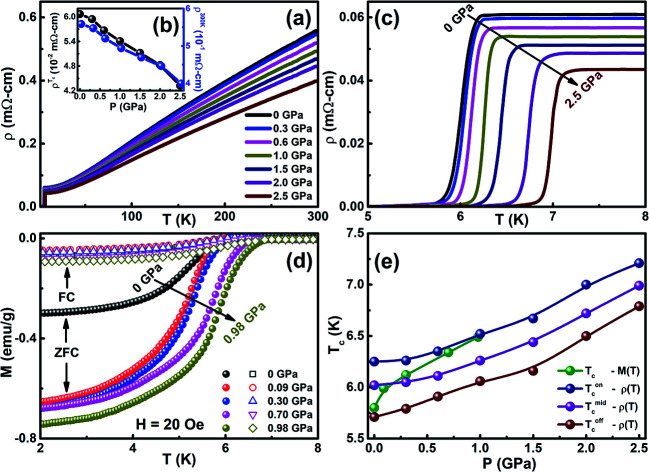

## Introduction

The interaction of superconductivity and density wave orders is an essential part of various superconductors such as high-*T*_c_ cuprates, Fe-based, BiS_2_-based, topological and two-dimensional (2D) layered superconductors.^1–8^ 2D transition metal dichalcogenide (TMDC) materials have attracted remarkable attention because of their exclusive properties that exhibit many features similar to those seen in high-*T*_c_ cuprates,^1,2^ MgB_2_,^9^ Fe-based^3,10^ and BiS_2_-based^4,5,11^ and 2D layered^7,8,12–15^ superconductors. The interplay of the static modulation of the electronic density of states close to the Fermi level and superconducting properties at lower temperatures in the layered TMDC has been of interest for a few decades.^7,8,12,13,16,17^ Most of these TMDCs have been built by three atomically dense layers stacked on each other like a sandwich that are held together by weak van der Waals forces, which results in a small coupling parameter between the layers. The spontaneous formation of periodic lattice distortions and charge density waves (CDWs) can be thermodynamically favorable under certain conditions in low dimensional metals, where the wave vector is generally known to depend on the nesting properties of the Fermi surface. Niobium diselenide (NbSe_2_) has one of the most studied van der Waals interactions, rendering it feasible to achieve a free-standing ultrathin structure that exhibits both CDW and superconductivity at low temperatures.^7,12^ The coupling between two long-range orders is responsible for the observation of the elusive Higgs bosonic amplitude mode of the superconductor, as revealed by Raman scattering on NbSe_2_.^18,19^ The crystal structure of TMDC materials is made up of hexagonal Se–Nb–Se sandwiches, which are united through weak forces, prominent hexagonal layers, and strong anisotropic properties. The changes in the electronic properties^20,21^ are produced by altering the lattice parameters,^22^ compositional tunings such as doping,^23,24^ irradiation^25,26^ and intercalation between the layers,^13,27–30^ and applying external hydrostatic pressures.^3,8,31,32^

The superconducting nature of TMDC has been studied intensively due to scarce magnetic and electronic properties and potential in technological applications. The superconducting properties can be modified by the intercalation of magnetic and non-magnetic impurities in NbSe_2_, such as M_*x*_NbSe_2_ (M = Cu, Fe, Co, Mn, Sn, Cr).^12,13,27–30^ The application of external pressure has been the most advantageous compared to other perturbations such as chemical doping, lattice disorder, impurity phases, and phase separation. The application of pressure fluctuates the interstitial distance and changes the electronic band structure of the material without providing any chemical stability. The variation in superconducting properties under pressure leads to lattice instability, which leads to the understanding of the superconducting mechanism of various materials such as high *T*_c_-cuprates, pnictides, and TMDC; these materials are most suitable to examine with theoretical models. By the application of external pressures (hydrostatic, quasi-hydrostatic, and uniaxial) to explore variation in physical properties and create new ground states for various types of matter. The uniaxial pressure effects evidently indicate that the in-plane compression is mainly accountable for reduction in the tilt angle and, hence, for the suppression of the pinning potential strength of cuprates.^33^ The enhancement in the superconducting nature with pressure has been reported with the highest *T*_c_ (164 K) in Hg-based cuprates^34,35^ and conventional superconducting *T*_c_ (203 K at *P* ∼200 GPa) induced by the application pressure in sulfur hydride systems.^36^ The application of external *P* is responsible for the increment in hole density in the superconducting layers of 11 and 122 families of pnictides.^10,37,38^ The effects of pressure on the superconducting and structural properties of oxypnictide compounds have been extensively explored.^3,39,40^ The external pressure induces the fluctuations in the Fermi surface (FS) with a positive pressure coefficient in NbS_2_.^41^ Hydrostatic pressure induces a transition from the spatial variation in the superconducting transition temperature pinning (*δT*_c_) to the spatial variation in the mean free path pinning (*δl*) mechanism in NbSe_2_ (ref. 8) and FeSe.^42^ The application of external *P* induces both *T*_c_ and CDW in TaS_2_ and TaSe_2_.^43^ The superconducting fluctuations such as enhancement in the superconducting *T*_c_, CDW instability, narrow superconducting transition width (Δ*T*), critical current density, and flux pinning properties are observed with the application of external *P* on TMDC compounds.^8,31,32,41,44,45^ Flux pinning is strongly related to vortex motion, which is an interesting physical phenomenon and plays a crucial role in practical applications.

Cr_*x*_NbSe_2_ is a type II superconductor and a versatile model system to study the interplay of magnetic interaction, superconducting, structural, and flux pinning properties at ambient and high pressures. In this present work, we investigated superconducting properties such as *T*_c_, upper critical field (*H*_c2_), lower critical field (*H*_c1_), irreversible field (*H*_irr_), superconducting critical current density (*J*_c_), and flux pinning of the superconducting Cr_*x*_NbSe_2_ single crystal at ambient and high pressures (∼1 GPa). Further, structural properties under ambient and high pressures at low temperature (9 K) were also investigated using synchrotron radiation to have a better understanding of the crystal structure and to correlate it with the superconducting properties.

## Experimental procedure

Single crystals of Cr intercalated NbSe_2_ were grown using the chemical vapor transport method with iodine as the transport agent. The magnetic, superconducting, and flux pinning properties of Cr_*x*_NbSe_2_ single crystals at ambient conditions have been recently reported.^27^ Structural measurements at ambient pressure were performed at X-ray diffraction (XRD) beamline BL-11, INDUS-II synchrotron radiation source (Raja Ramanna Centre for Advanced Technology, Indore, India) with Angle Dispersive X-ray Diffraction (ADXRD) beamline (BL-11). The samples were powdered, drop cast on copper-coated carbon grids after sonication in iso-propyl alcohol for 1 h, and air-dried for 5 h. Microstructural analyses of the sample were performed using a Tecnai G2 30ST (FEI) transmission electron microscope. Both temperature and field dependence of DC magnetization measurements (*M*(*T*) & *M*(*H*)) under various hydrostatic *P* was carried out using Physical Property Measurement System-Vibrating Sample Magnetometer (PPMS-VSM, Quantum Design, USA). The external *P* of up to ∼1 GPa was generated in the clamp type miniature hydrostatic pressure cell, which was made of non-magnetic Copper–Beryllium alloy. Fluorinert FC #70 and FC #77 mixture (1 : 1) was used as a pressure transmitting medium and *in situ* pure Sn was loaded with the sample in a capsule; actual *P* was calculated from the *M*(*T*) measurements of Sn under various *P* at 10 Oe. Electrical transport measurements were carried out in PPMS using the standard four-probe technique both at ambient and high pressure up to ∼2.5 GPa. The pressure cell consists of a double-wall cylinder, which is made of hardened BeCu (outer) and NiCrAl (inner) alloy, and the obturator is made of hardened BeCu alloy. Daphne #7474 was used as the pressure medium and the pressure cell was calibrated with the Bi-phase transitions at room temperature.

To understand the valence band (VB) evidence of the Cr_*x*_NbSe_2_ single crystalline sample, ultraviolet photoelectron spectroscopy (PES) measurements were performed at the Angle-Resolved Photoelectron Spectroscopy beamline in BL-3, INDUS-II with synchrotron radiation source (RRCAT, Indore, India). Raman measurements at low temperature were carried out using LABRAM HR-800 spectrometer from Horiba JY, Japan equipped with a 488 nm excitation source, an 1800 g mm^−1^ grating, and a CCD detector; temperature stability of ±1 K was maintained during the measurements. Structural measurements under high pressure at room temperature were performed at the ADXRD beamline BL-11, INDUS-II synchrotron radiation source (RRCAT, Indore, India). Powder X-ray diffraction measurements under high pressure at room temperature were carried out using a Mao Bell-type diamond anvil cell (DAC). A pre-indented thickness of 0.2 mm Cu material served as the gasket, a mixture of methanol–ethanol (4 : 1) was used as the pressure medium, and the sample chamber was filled with both Cr_*x*_NbSe_2_ powder sample and a ruby chip. The ADXRD patterns were measured using a beam wavelength of 0.4828 Å with a sensitive detector (Mar3450). The actual *P* in DAC was measured using the ruby fluorescence technique.

## Results and discussion

**Fig. 1:**
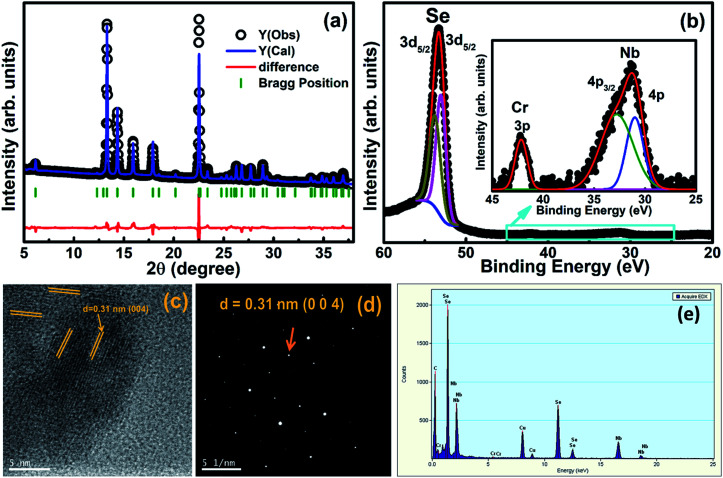
(a) Powder X-ray diffraction was measured at room temperature using synchrotron radiation source, (b) photoelectron spectrum at 300 K recorded at 100 eV photon energy with a synchrotron source, (c) HRTEM image shows the stacking of (004) planes corresponding to the NbSe_2_ crystal structure, (d) SAED pattern with (004) reflection marked, and (e) EDX analysis shows the presence of Se, Nb, Cu, C, and Cr signals coming from the copper-coated carbon grid of the Cr_0.0009_NbSe_2_ single crystal.

Powder X-ray diffraction (XRD) patterns were measured using the synchrotron energy source as shown in Fig. 1(a). The XRD pattern at room temperature was refined using the Rietveld refinement method and it confirms the hexagonal crystalline phase with a space group of *P*6_3_/*mmc*. The lattice parameters were estimated with pseudo-Voight function for peak profile fitting using FullProf software^46^ and the obtained lattice parameter values (300 K) are *a* = *b* = 3.4432(5) Å, *c* = 12.5467(3) Å, and *V* = 128.825 Å^3^ with fitting parameters *χ*^2^ = 3.493, *R*_wp_ = 0.05, and *R*_p_ = 0.07. Further, the XRD patterns confirmed that there is no impurity peak and secondary phase due to Cr intercalation in NbSe_2_. Photoelectron spectroscopy (PES) was performed using a synchrotron radiation source (100 eV photons) at room temperature and 3d core levels of Se, 4p level of Nb, and 3p level of Cr were measured after subtraction of background using the standard Shirley method, as shown in Fig. 1(b).^47^ The binding energy of Se 3d_5/2_ and 3d_3/2_ peaks are located at ∼53.05 and ∼53.8 eV, respectively, and designate the occurrence of two most important Se species peaks separated by ∼0.75 eV, when deconvoluted using typical constrained parameters. The peak binding energies of ∼30.99 and ∼32.77 eV originate from Nb 4p_1/2_ and 4p_3/2_, respectively,^48,49^ and the Cr 3p level is observed at ∼42.16 eV.^50^ These results confirm the presence of Nb, Se, and Cr elements in the as-grown single crystal. The stacking of (004) planes of the NbSe_2_ crystal structure (ref: JCPDS card no. 01-070-5612, 72-0864, and 72-1618) can be easily identified in the HRTEM images (Fig. 1(c)). The interplanar distance corresponds to the (004) plane, which was calculated to be approximately 0.31 nm, using the ImageJ software. This was further established both by the corresponding Fast Fourier Transform (FFT) and Selected Area Electron Diffraction (SAED) patterns. The SAED pattern clearly shows the single-crystal diffraction characteristics with sharp and discrete Bragg reflections. The characteristic Bragg reflections corresponding to the NbSe_2_ crystal structure could be identified in the SAED pattern, as shown in Fig. 1(d). The EDX results show the presence of Cr, Nb, Se, Cu, and C, where the signals of Cu and C are due to the copper-coated carbon grid and the signals of Cr, Nb, and Se are from the sample (Fig. 1(e)). Quantitative analysis shows the atomic percentage of Cr, Se, and Nb in the sample to be 0.10%, 64.96%, and 34.93%, respectively; the elemental compositions are present in the stoichiometric ratios of the as-grown crystals and no impurity was found in this sample.

**Fig. 2:**
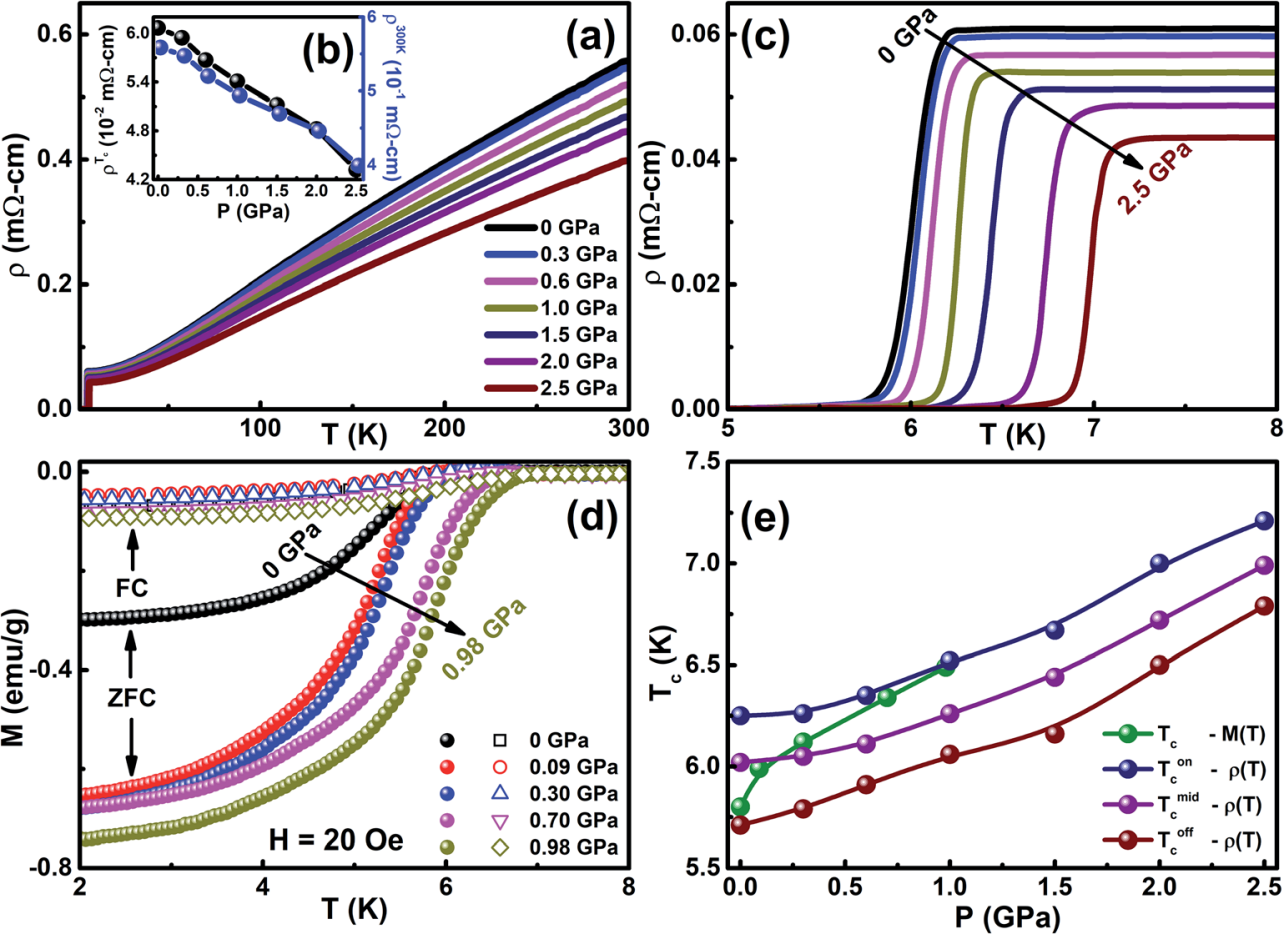
(a) Temperature dependence of resistivity (*ρ*(*T*)) under various hydrostatic pressures up to ∼2.5 GPa, (b) pressure dependent resistivity at *T*^on^_c_ and 300 K, (c) enlarged temperature region near *T*_c_ from *ρ*(*T*) under pressures up to 2.5 GPa, (d) temperature dependent dc magnetization measurements (*M*(*T*)) in zero-field cooling (ZFC-solid symbol) and field cooling (FC-open symbol) at *H* = 20 Oe near *T*_c_ under various pressures up to ∼1 GPa, and (e) pressure dependent superconducting transition temperatures (*T*_c_, *T*^on^_c_, *T*^mid^_c_, and *T*^off^_c_) for Cr_0.0009_NbSe_2_.

Fig. 2(a), (c), and (d) show the temperature dependence of resistivity (*ρ*(*T*)), superconducting region in the temperature region 5 to 8 K at various hydrostatic *P* up to ∼2.5 GPa, and dc magnetization (*M*(*T*)) in zero-field cooling and field cooling at *H* = 20 Oe under ambient and high pressures up to ∼1 GPa in the vicinity of *T*_c_ on the Cr_0.0009_NbSe_2_ single crystal. We found that the in-plane resistivity decreases from 0.56 mΩ cm to 0.061 mΩ cm as temperature decreases from 300 to 6 K and then drops suddenly to zero due to the manifestation of superconductivity, and the residual resistance ratio (RRR = *ρ*_300 K_/*ρ*_*T*_c__) is 9.2, which indicates that the quality of the single-crystal sample is good. The superconducting *T*_c_ from *ρ*(*T*) measurements is defined using different criteria such as *T*^on^_c_, *T*^mid^_c_, and *T*^off^_c_. The onset of superconducting transition temperature (*T*^on^_c_) is determined from the intersection of two extrapolated lines; one is drawn through the resistivity curve in the normal state just above the appearance of superconductivity and the other line is drawn through the sheer part of the resistivity curve in the superconducting state. The midpoint of superconducting transition (*T*^mid^_c_) is estimated from the peak position of d*ρ*/d*T vs. T*, *i.e.*, the temperature at which *ρ* displays the maximum value and the offset of superconducting transition (*T*^off^_c_) is estimated from the temperature at which resistance becomes zero. The superconducting transition width (Δ*T* = *T*^on^_c_ − *T*^off^_c_) was found to be 0.54 K with narrow Δ*T*, reflecting the virtuous quality of the as-grown single crystal. The observation of the narrow superconducting width of *T*_c_ and zero resistance indicates that the hydrostatic nature is good in our resistivity experiments. Fig. 2(c) shows a very sharp transition at 5.8 K at ambient pressure, where diamagnetism starts appearing and it confirms the bulk superconducting nature, which is in good agreement with the *ρ*(*T*) measurements of the same sample. The superconducting nature of *T*^on^_c_, *T*^mid^_c_, and *T*^off^_c_ are enhanced by external pressure, as shown in Fig. 2(e). The rate of change of *T*^on^_c_ with *P*(d*T*_c_/d*P*) up to ∼1 GPa is 0.27 K GPa^−1^ at less than 1 GPa pressure and is 0.46 K GPa^−1^ in the pressure range of 1 ≤ *P* ≤ 2.5 GPa, which is observed from the *ρ*(*T*) measurements. Normal state resistivity was found to gradually decrease with external hydrostatic *P*, as shown in Fig. 2(b), and it exhibits metallic nature in the entire pressure region up to ∼2.5 GPa. The similar nature of *ρ*(*T*) under high *P* ∼2.5 GPa has been reported in various superconducting materials.^31,32,35,38–40,43–45^ This is related to the fact that the application of *P* brings the layers close together and simplifies the overlap of wave functions of the conduction electrons in the neighboring layers. The *T*_c_ observed from the magnetization measurements is less than the *ρ*(*T*) measurements and such a difference in *T*_c_ has been reported in various superconducting samples. The sample exhibits a sturdy diamagnetic signal and the high superconducting shielding fraction (∼82%) represents that bulk superconductivity is exhibited by Cr intercalated NbSe_2_. The *T*_c_ of Cr_*x*_NbSe_2_ is found to be less than that of pure NbSe_2_ (ref. 8 and 13) and the analogous tendency of superconducting properties under both ambient and high pressure have been found in various intercalated compounds such as M_*x*_NbSe_2_ (M = Fe, Cr, Sn, Cu, Al),^12,13,22,27,29^ Sr_0.1_Bi_2_Se_2_,^6,51^ and Fe-based superconductors.^52–55^Fig. 2(d) shows the temperature dependence of dc magnetization (*M*(*T*)) in zero-field cooling (shielding) and field cooling (Meissner) with an external magnetic field of 20 Oe under various *P* up to ∼1 GPa for Cr_0.0009_NbSe_2_. The values of the shielding and Meissner signal are enhanced by the applied *P* and it is shown in Fig. 2(d). The external pressure increases the superconducting nature and it reveals the strong enhancement in the pinning potential. The onset of SC transition temperature (*T*_c_) is found from the ZFC curve shifts towards higher temperature by the application of hydrostatic pressure. The external pressure influences the Fermi surface topological fluctuations that seem to be responsible for the enhancement in *T*_c_ and it is consistent with the previous report.^20^ On increasing the *P* up to 0.98 GPa, it was found that both *T*_c_ and the superconducting shielding fraction increase gradually up to 0.98 GPa, as shown in Fig. 2(e). We defined the onset of diamagnetic shielding fractions at 5.8 K (0 GPa) and 6.76 K (0.98 GPa) and the pressure dependence of *T*_c_ are shown in Fig. 2(e). It is found that d*T*_c_/d*P* = 0.98 K GPa^−1^ and it is higher than the *ρ*(*T*) measurements. *T*_c_ enhancement under *P* in Cr_*x*_NbSe_2_ is found to be larger than the *P* dependence of *T*_c_ reported for other 2D layered metallic superconductors,^8,31,32,41^ such as BiS_2_-based^4^ and Fe-based superconductors.^3^ The application of *P* leads to a reduction in the interlayer distance, which is responsible for the enhancement in the superconducting nature of TMDC compounds. Further, the reduction in the interlayer distance under pressure paves the way for an increase in the electron–phonon coupling constant, which leads to broadening of the energy bands, thus providing a dominant contribution to the enhancement in *T*_c_. These results imply that an increase in the density of states (DOS) at the Fermi level is due to the application of *P*. The electronic structures have been dominated by the hybridization between the transition metal 4d and the Se 4p orbital, which is accountable for the covalent Se–Nb–Se bonds. The hybridization is very weak in Se 4p and d electrons in transition metal ions, which confirms the formation of the Cr–Se bond.^20,21^ The Fermi level is primarily contributed by d electrons of Nb and intercalated transition metal ions, which are responsible for the metallic character and give a better understanding of intercalated NbSe_2_ compounds. The external *P* effect on the superconducting properties of the 2D layered NbSe_2_ compounds was characterized by the fact that the compressibility is seven times larger perpendicular to the layers than parallel to the layers^44^ and hence, it is causes the layers to move close together. The application of *P* suppresses the lattice instability with simultaneous increase in *T*_c_ by 1.4 K GPa^−1^, as shown in the phase diagram *P*(*T*_c_) (Fig. 2(e)). The lattice instabilities increase under *P*, which eventually increases the electron–phonon interactions and leads to an increase in *T*_c_ from both *ρ*(*T*) and *M*(*T*) in Cr_*x*_NbSe_2_ systems.

We analyzed the *ρ*(*T*) measurements under various *P* by analyzing the Fermi-liquid model, *ρ*(*T*) = *ρ*_0_(*T*) + *AT*^2^, where *ρ*_0_ and *A* are the residual resistivity and electron–phonon scattering factor, respectively, in the temperature range of 10 < *T* < 50 K. The pressure dependent values of *ρ*_0_ and *A* are shown in Fig. 4(a) and it is clearly indicated that both are reduced by the application of *P*. As seen from the *ρ*(*T*) curve in the low temperature region under various pressures, the Fermi-liquid nature is followed. *ρ*(*T*) in the 10 to 50 K range is invariably linear for all *P*, arising entirely from the electron–acoustic phonon interaction. This is the general behavior of *ρ*(*T*) due to the presence of phonons in all types of superconducting materials. In the framework of Fermi-liquid theory, factor *A* is proportional to the charge carrier effective mass. The suppression of the scattering factor with external pressure suggests that the reduction in effective mass leads to a gradual loss of electron correlation with the enhancement in superconductivity. The temperature dependent upper critical field *H*_c2_(*T*) is determined from the onset of Meissner signal observed from *M*(*T*) with various *H* yields in the *H*_c2_–*T* phase diagram fitted with the Ginzburg–Landau function, *H*_c2_(*t*) = *H*_c2_(0)((1 − *t*^2^)/(1 + *t*^2^)), where *t* = *T*/*T*_c_, as shown in Fig. 4(b). The orbital limit upper critical field of type II superconductors was estimated using Werthamer–Helfand–Hohenberg (WHH) theory in dirty limit, which is *H*^orb^_c2_(0) = −0.693*T*_c_(d*H*_c2_/d*T*)_*T*_c__.^56^ Beneath the orbital limiting case, pair breaking takes place due to an increase in the kinetic energy of the Cooper pair, which are comparable to the condensate state in the presence of both external magnetic field and hydrostatic pressure. In Pauli paramagnetic limit, the spin alignment with the application of magnetic field that favors energy conditions leads to the breaking of Cooper pairs. According to the weak coupling case, the Pauli limited upper critical field is given by *H*_p_(0) = 1.84*T*_c_, which indicates the influence on both the pair breaking mechanism and Pauli spin paramagnetic effect. The upper critical fields (*H*^orb^_c2_(0), *H*_c2_(0), & *H*_P_(0)) are found to increase with the application of *P* and it is given in Table 1. The enhancement in the upper critical fields under various *P* indicates that strong flux pinning is exhibited in Cr intercalated NbSe_2_ single crystal. The Maki parameter measures the relative strengths of the orbital and Pauli limiting fields are calculated using the relation 
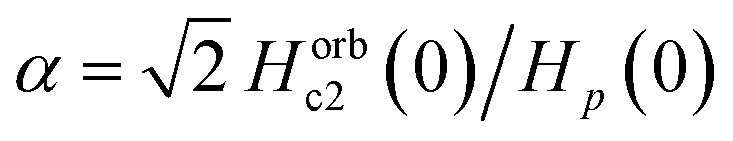
. The Maki parameter was found to be 1 for both ambient and high *P*, which indicates that bulk superconductivity is Pauli limited (*H*_c2_(0) < *H*^orb^_c2_(0) < *H*_p_(0)); the Flude–Ferrell–Larkin–Ovchinnoikov (FFLO) state, which is insightful of the spatially moderated order parameter, is not favorable.^57^ The coherence length is calculated from the Ginzburg–Landau expression as *H*_c2_(0) = *ϕ*_0_/2π*ξ*(0)^2^, where *ϕ*_0_ = 2.07 × 10^−7^ G cm^2^ and the estimated values are listed in Table 1. The value of *α* increases with *P*, which indicates that the Pauli spin paramagnetic effect drastically increases as external *P* is increased.

**Table 1:** Superconducting parameters *H*_c2_(0), *H*^orb^_c2_(0), *H*_p_(0), *H*_c1_(0), *λ*(0), and *ξ*(0) of Cr_*x*_NbSe_2_

*P* (GPa)	*H*_c2_(0) (T)	*H* ^orb^_c2_(0) (T)	*H*_p_(0) (T)	*H*'_c1_(0) (mT)	*H*_irr_(0) (T)	*ξ*(0) (nm)	*λ*(0) (nm)
0	4.5 ± 0.02	4.4 ± 0.05	9.90	51 ± 0.5	0.85 ± 0.01	8.6 ± 0.10	51.01 ± 0.01
0.30	6.7 ± 0.05	6.0 ± 0.03	11.20	49 ± 0.3	1.35 ± 0.02	7.0 ± 0.05	48.76 ± 0.02
0.98	8.4 ± 0.03	7.3 ± 0.01	12.40	47 ± 0.8	1.46 ± 0.01	6.3 ± 0.10	46.98 ± 0.01

**Fig. 3:**
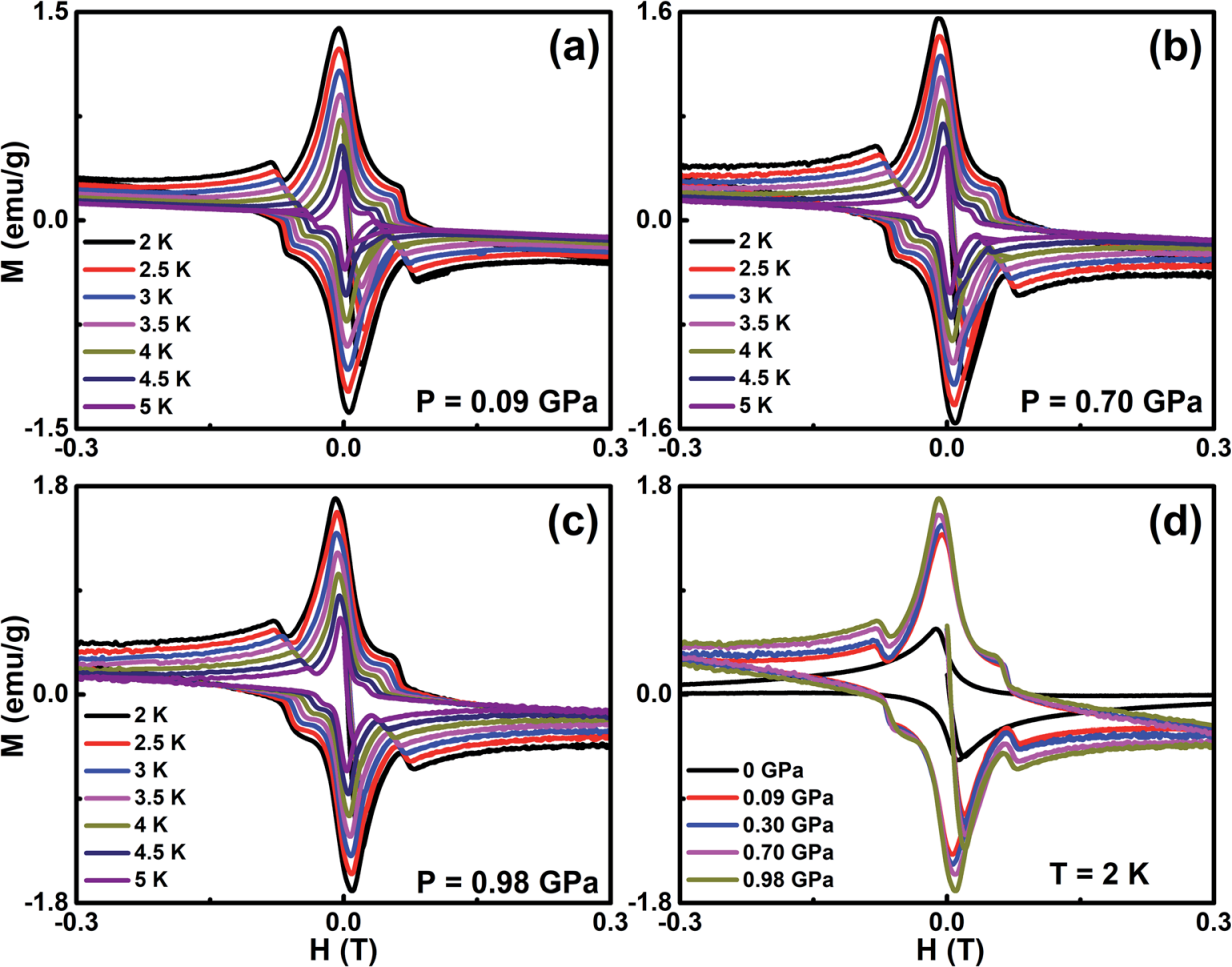
(a–c) Field dependent magnetization (*M*(*H*)) under the pressure of 0.09 GPa, 0.70 GPa, and 0.98 GPa in the temperature range from 2 K to 5 K with the interval of 0.5 K, and (d) field dependent magnetization (*M*(*H*)) with a slow scan rate of 20 Oe s^−1^ at 2 K at the selective pressure of 0 GPa, 0.09 GPa, 0.30 GPa, 0.70 GPa, and 0.98 GPa.

**Fig. 4:**
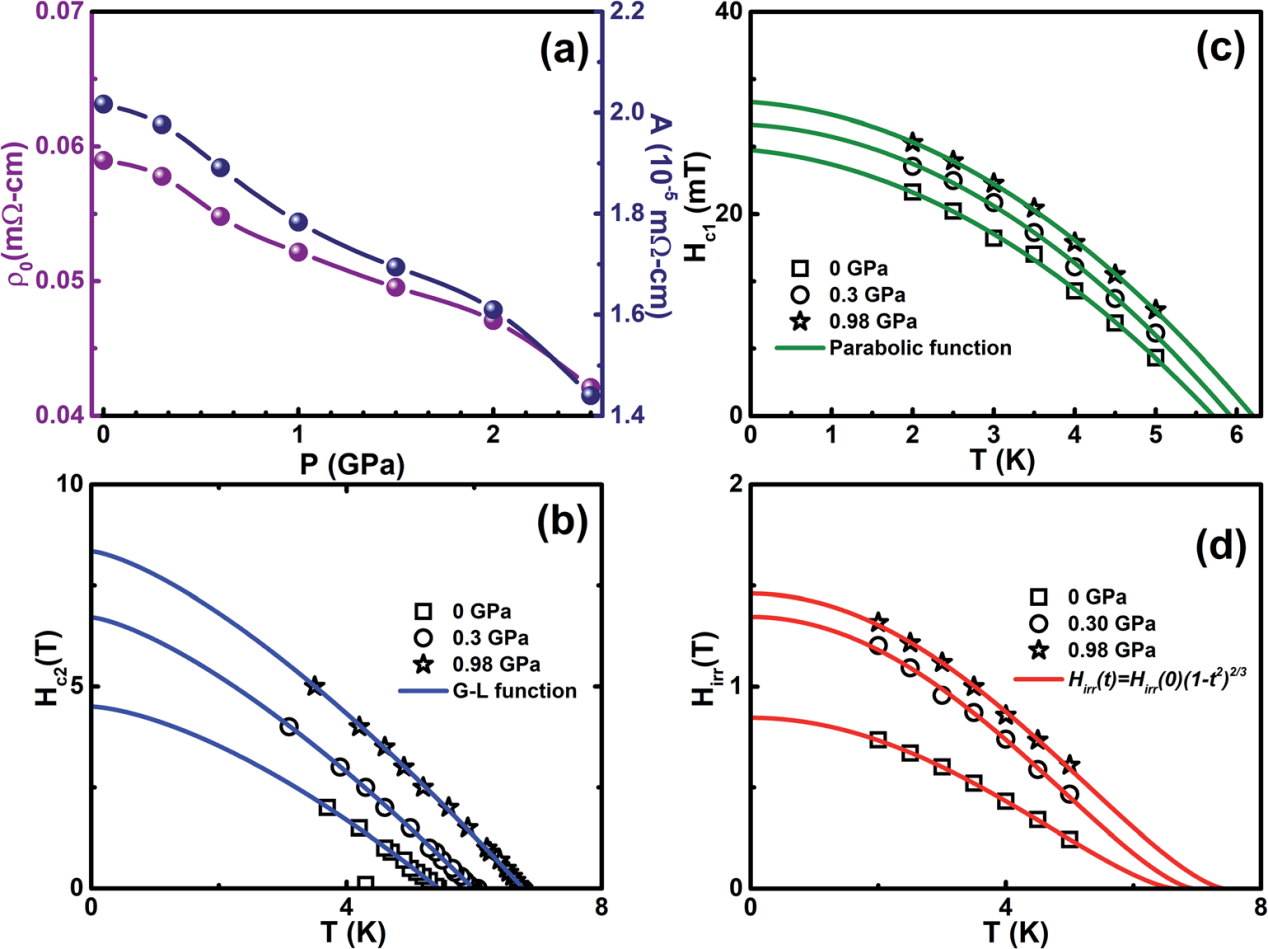
(a) Pressure dependence of residual resistivity (*ρ*_0_) and scattering factor (*A*) extracted from the fitting the relation, *ρ*(*T*) = *ρ*_0_(*T*) + *AT*^2^ in the *ρ*(*T*) curve, (b) upper critical field (*H*_c2_) as a function of temperature at selective *P* and the solid lines represent the Ginzburg–Landau function, (c) temperature dependence of lower critical field (*H*_c1_) at selective *P* and the solid lines are fitted with *H*_irr_(*t*) = *H*_irr_(0)(1 − *t*^2^)^3/2^, and (d) temperature dependence on the irreversible field (*H*_irr_) under selective *P* and the solid lines represent the parabolic function fitting for Cr_0.0009_NbSe_2_.

We measured the magnetic field dependent magnetization (*M*(*H*)) below the superconducting state under various *P* and it reveals the linear variation of magnetization, which is a signature of the Meissner state and it is clearly seen in the low field region, as shown in Fig. 3. At ambient pressure, there are no additional peaks in the isothermal magnetization curves for Cr_0.0009_NbSe_2_.^27^ The application of pressure isothermal magnetization exhibits a small magnetization peak close to 0.1 T below the superconducting transitions and it is known as the second magnetization peak (SMP). The isothermal magnetization curves (Fig. 3) show a kink in the field close to 0.1 T on the application of pressure, which is called as the second magnetization peak. This is the first time that we observed SMP behaviour in 2D layer superconductors and we recently reported a similar behaviour in Fe intercalated NbSe_2_.^8^ The SMP behaviour exists in few selective superconductors (cuprates and Fe based superconductors) in a limited temperature range below *T*_c_.^58,59^ The doping effect also induces the SMP behaviour and it has been observed in cuprates and Fe based superconductors.^58,59^ The lower critical field (*H*_c1_(*T*)) is estimated by a deviation from linearity in the diamagnetic state of the *M*(*H*) curve, as shown in Fig. 3. Above this field, Abrikosov vortices become energetically favorable and start entering the sample edges.^60^*H*_c1_(*T*) can be described in terms of the monotonic curve with a quadratic dependence, which is dictated by the empirical relation, *H*_c1_(*T*) = *H*_c1_(0)(1 − (*T*/*T*_c_)^2^) and shown in Fig. 4(c). Further, the experimental data with G–L fitting suggests that the vortex that penetrates into this sample could be well described by BCS theory. The deflection of field lines around the sample leads to a more pronounced Meissner slope given by *M* = −*H*_*a*_/(1 − *N*), where *N* is the demagnetization factor. Although the demagnetization effect is very small, the corrected value of the lower critical field 
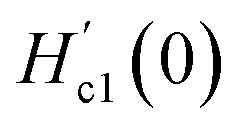
 after demagnetization correction is incorporated in *H*_c1_(0) by using Brandt's formula,^61^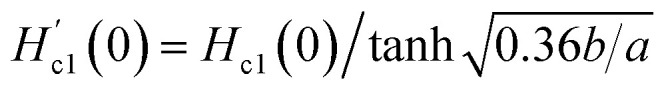, where *a* and *b* are the breadth and thickness of the samples, and the calculated values are listed in Table 1. *H*_c1_(0) is related to two fundamental length scales such as the London penetration depth (*λ*(0)) and the coherence length (*ξ*(0)). According to the Ginzburg–Landau relation, 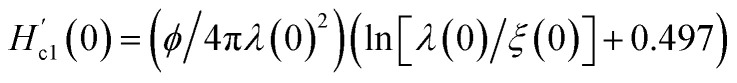 (ref. 62) where *λ*(0) and *ξ*(0) give the G–L parameter (*κ*) expressed by the relation, *κ* = *λ*(0)/*ξ*(0). The critical value of *κ* is 
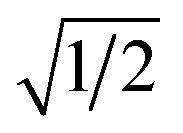
. which is conventionally classified into type I and type II superconductors. The value of *κ* larger than the critical value designates the type II superconductors as a robust type. The thermodynamic critical field (*H*_c_) is obtained from the relation, *H*_c1_(0)*H*_c2_(0) = *H*_c_^2^ ln(*λ*(0)/*ξ*(0)) and the values are 256 mT (0 GPa), 316 mT (0.3 GPa), and 359 mT (0.98 GPa). The magnetization curves indicate that the melting of vortices start at field *H* > *H*_irr_, which is smaller than the upper critical field. A similar nature is typically observed in high-*T*_c_ superconductors, where melting of vortices is accredited to thermal fluctuations and is not often exhibited in low *T*_c_ superconductors. The calculated values of *λ*(0) and *ξ*(0) at ambient and high *P* are listed in Table 1. The irreversible field (*H*_irr_) estimated from *M*(*H*) using Kramer's plot (*H vs. J*_c_^0.5^*H*^0.25^) indicated that the depinning of magnetic flux pinning occurs within the *H*_irr_ of the superconducting sample. Fig. 4(d) shows the extrapolation of the H-T phase diagram fitted with the parabolic function *H*_irr_(*T*) = *H*_irr_(0)(1 − (*T*/*T*_c_)^2^)^3/2^ in the *P* range of 0 to 1 GPa. It shows the enhancement in *H*_irr_ with the application of *P* and it is influenced by flux pinning. The extrapolated values of *H*_irr_(0) at ambient and high pressures are tabulated in Table 1. All these may robustly propose the existence of the quantum vortex state due to quantum instability of the vortices.

**Fig. 5:**
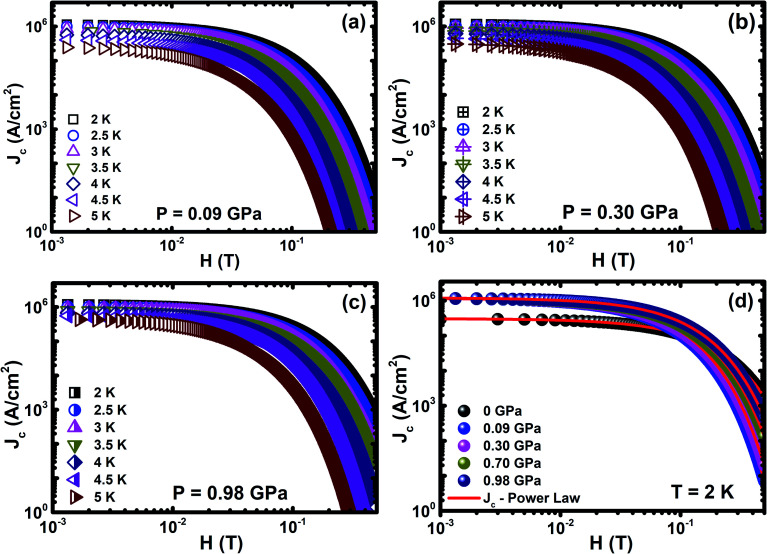
(a–c) *J*_c_(*H*) for selected pressures of 0.09 GPa, 0.30 GPa, and 0.98 GPa under various temperatures and (d) *J*_c_(*H*) for various pressures up to ∼1 GPa at the constant temperature of 2 K.

The magnetic field dependence of critical current density (*J*_c_(*H*)) at various temperatures is estimated from the magnetic hysteresis loop (*M*(*H*)) using Bean's model under various pressures up to ∼1 GPa, as shown in Fig. 5. The *M*(*H*) curve exhibits both bulk superconductive and vortex pinning nature in the Cr_*x*_NbSe_2_ single crystal. Unquestionably, the observed demagnetization is not due to the superconducting surface shielding fraction effect and it could be understood using the Bean critical state model.^63^ For the rectangle sample, *J*_c_(*T*, *H*) = 20Δ*m*(*T*, *H*)/(*ω*^2^(3*l* − *ω*)), where Δ*m*(*T*, *H*) is the separation between the two branches of *M*(*H*), and *l* and *ω* are the length and width of the sample (*l* > *ω*), respectively. At lower magnetic fields, the width of the magnetic moment (Δ*m*(*T*, *H*)) is essentially caused by the inter-granular current. The estimated *J*_c_ exponentially decreases with the application of external *H* and it seems to be correlated with weak *H* dependence of the pinning potential. The weak dependence of *J*_c_ on *H* and *T* suggests that the Cr_0.0009_NbSe_2_ single crystal has a greater *J*_c_ behaviour, which is beneficial for potential application in low and high *H*. *P* enhances *J*_c_ in the sample and consequently increases the vortex dynamics properties, which leads to the enhancement in point pinning centers. The values of *J*_c_(0) at 2 K under various *P* were found to be are 304 732 (0 GPa), 1 180 204 (0.09 GPa), 1 209 901 (0.30 GPa), 1 214 477 (0.70 GPa), and 1 247 649 (0.98 GPa) A cm^−2^, which are larger than the parent NbSe_2_.^13^ Further, *J*_c_ increases four-fold with the increase in *P* (∼1 GPa) compared with the ambient *P*. However, *J*_c_(*H*) was found to decrease moderately at a low field and it shows a sharp decrease in higher fields. From collective theory,^64^ the exponential law by the relation follows as *J*_c_(*H*) = *J*_0_ exp(−(*H*/*H*_0_)^3/2^) under various *P*, where *J*_0_ and *H*_0_ are the zero field *J*_c_ and normalization parameter, respectively, and it is shown in Fig. 5(d). The divergence at low magnetic fields is associated with the crossover from single-vortex pinning regime to the small bundle pinning regime. The high field departure that is very close to the irreversibility field (*H*_irr_) line could be associated with large thermal fluctuations, a view that is supported by the three-dimensional (3D) flux creep reliance observed for the dissimilarity of *H*_irr_, as shown in Fig. 4(d). The temperature dependent *H*_irr_ at various *P* is linearly extrapolated using Kramer's plot (*H vs. J*_c_^0.5^*H*^0.25^).^65^ The high field peculiarity that is very close to the irreversibility line could be associated with huge thermal instabilities, a view that is supported by the 3D flux creep dependence detected for the variation in field dependence of *J*_c_ at various temperatures and pressures.

**Fig. 6:**
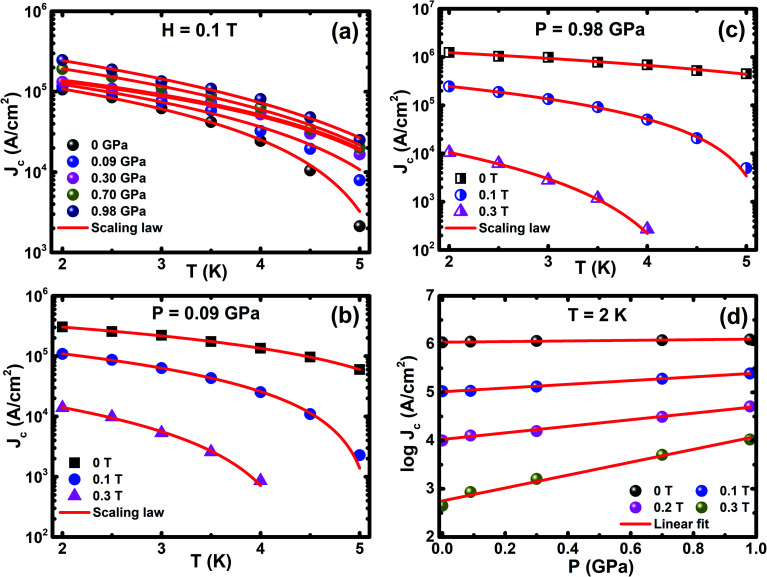
(a) *J*_c_(*T*) at 0.1 T under various *P* and it is fitted with the relation *J*_c_(*T*) ∝ (1 − (*T*/*T*_c_))^*a*^, (b) *J*_c_(*T*) at 0.09 GPa under various magnetic fields, (c) *J*_c_(*T*) at 0.98 GPa under selected magnetic fields and (d) *J*_c_(*P*) at 2 K; the solid lines represent linear fitting.

*J*_c_ is connected with thermally activated flux flow, which is explained by the empirical formula, *J*_c_(*T*) ∝ (1 − (*T*/*T*_c_))^*β*^, where *β* is a critical exponent. The value of the *β* parameter denotes the distinct vortex pinning mechanism in superconducting materials and it is consistent with the G–L theory. From the G–L theory, the critical exponent is used to classify the vortex pinning mechanisms at precise magnetic fields. The value *β* = 1 indicates non-interacting vortices and *β* ≥ 1.5 signifies the core pinning mechanism.^66^ The various values of *β* have been found for transition metal intercalated NbSe_2_, which illustrate different core pinning mechanisms under ambient and high pressures.^8,13,27,31^ These empirical relations estimate the temperature dependence of various critical parameters and reveal the freezing out of quasiparticle excitations by the BCS energy gap. The critical exponent is estimated from fitting the scaling relation, as shown in Fig. 6, under various magnetic fields and external *P*. It reveals that the values of *β* are found to be 1.59 ≤ *β* ≤ 1.96 (0 T) and 2.32 ≤ *β* ≤ 2.81 (0.1 T) under various *P* up to ∼1 GPa and the *β* values observed under various *P* is larger than that under ambient *P*, which demonstrates the vigorous enhancement in current density with pressure. Fig. 6(d) shows *J*_c_ dependence of *P* at 2 K under various *H* and the solid lines show the linear fits to the experimental data, which gives the slopes (*d*(log *J*_c_(*T*, *H*))/d*P*) of 0.06, 0.39, 0.68, and 1.34 GPa^−1^ at 0, 0.1, 0.2, and 0.3 T, respectively. The striking interface between the vortices and the pinning centres prevent the movement of vortices in type II superconductors. These results indicate that the application of *P* leads to enhancement in *J*_c_ and it helps to understand the pinning mechanism in this sample.

**Fig. 7:**
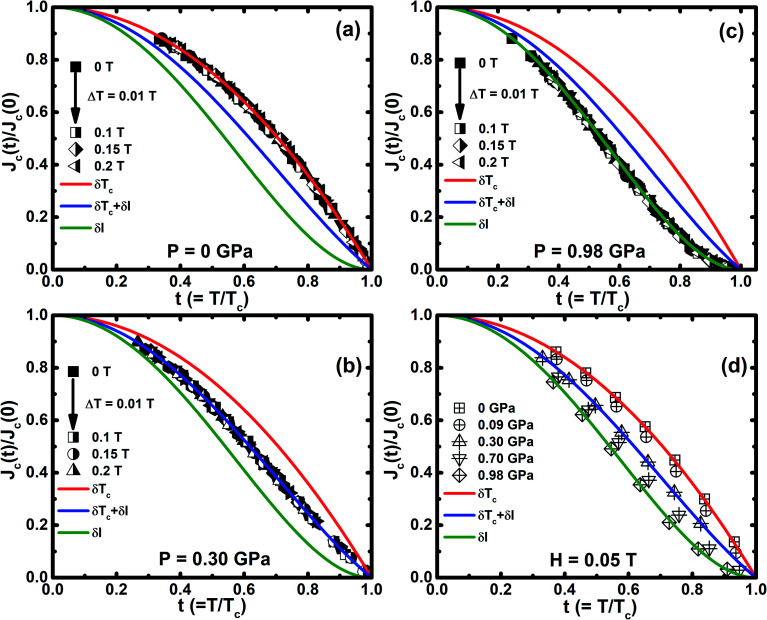
(a–c) Reduced temperature (*t* = *T*/*T*_c_) dependence of normalized *J*_c_(*J*_c_(*t*)/*J*_c_(0)) for the selected *P* of 0 GPa, 0.30 GPa, and 0.98 GPa in the magnetic field range from 0 to 0.2 T, and (d) *J*_c_(*t*)/*J*_c_(0) as a function of reduced *T*/*T*_c_ at *H* = 0.05 T for various *P* up to ∼1 GPa. Continuous lines denote the theoretical curve fitting, which are fitted based on the model: *δT*_c_ (red curves), *δl* (green curves), and coexistence of both *δT*_c_ + *δl* (blue curves) pinning mechanisms.

The flux pinning is reflected in *J*_c_(*H*) and *J*_c_(*T*) with the application of *P*, as shown in Fig. 7, and the pinning mechanism varies with the application of *P* at the critical pressure (*P*_c_) of 0.3 GPa. *J*_c_(*T*) is well explained in the framework of the model of the collective flux pinning and creep.^64^ For type II superconductors, the vortices interact with the pinning centers through spatial fluctuations in *T*_c_(*δT*_c_ pinning) with *h*_max_ ∼0.67, 0.6, and 0.5 for point, surface, and body pinning, respectively. The vortices interact by the scattering of charge carriers with *l* (*δl* pinning) with *h*_max_ ∼0.33 and 0.2 corresponding to the point and surface pinning, respectively. The external pressure increases both the dislocation and deformation stress in Cr_*x*_NbSe_2_. These dislocations might interact with one vortex line when the dislocation is parallel to the local field with several vortex lines when there is an angle that exists between the dislocation and the local field. Consequently, we believe that the extensively occurring dislocations also contribute to the vortex pinning. The normalized *J*_c_ as a function of reduced temperature (*t* = *T*/*T*_c_) is described by *δT*_c_-pinning (*J*_c_(*t*) ∝ (1 − *t*^2^)^7/6^(1 + *t*^2^)^5/6^) for less than *P*_c_, when *T*_c_ fluctuates due to both Cr intercalation and point defects (Fig. 7(a)), which are the foremost sources for trapping the vortices. Further, *J*_c_(*t*) shows completely different behavior for *P* ≥ *P*_c_, which leads to an increase in *J*_c_, followed by a crossover from *δT*_c_-pinning to *δl*-pinning (*J*_c_(*t*) ∝ (1 − *t*^2^)^5/2^(1 + *t*^2^)^−1/2^) at 0.98 GPa with various magnetic fields up to 0.2 T are shown in Fig. 7(c) and a similar nature has been observed by us in the parent NbSe_2_.^8^ A similar crossover occurs from *δT*_c_ to *δl* pinning and it has been reported in NbSe_2_ and FeSe, and this phenomenon occurs due to both chemical doping^13^ and application of external hydrostatic pressure.^8,42^ This suggests that spatial fluctuation in the mean free path (*l*) of the charge carrier becomes crucial for flux pinning above *P*_c_. From the above results that indicate the intercalated non-magnetic (Cr), it was found that the impurity is not uniformly distributed in the layered structure of NbSe_2_, which leads to the random distribution of dislocations. By the application of external *P*, the decrease in the interlayer distance prompts the formation of dislocations in the Se–Nb–Se layers. The enlarged fluctuation in *l* is due to the variation in coherence length (*ξ*) with external *P*. This implies that the disorder parameter characterizes the collective vortex pinning, which is proportional to *ξ* and to 1/*ξ*^3^ for *δT*_c_ and *δl*-pinning, respectively. *H*_c2_(0) is enhanced by the application of *P*, indicating that the variation in *ξ* is possibly significant for the crossover.

**Fig. 8:**
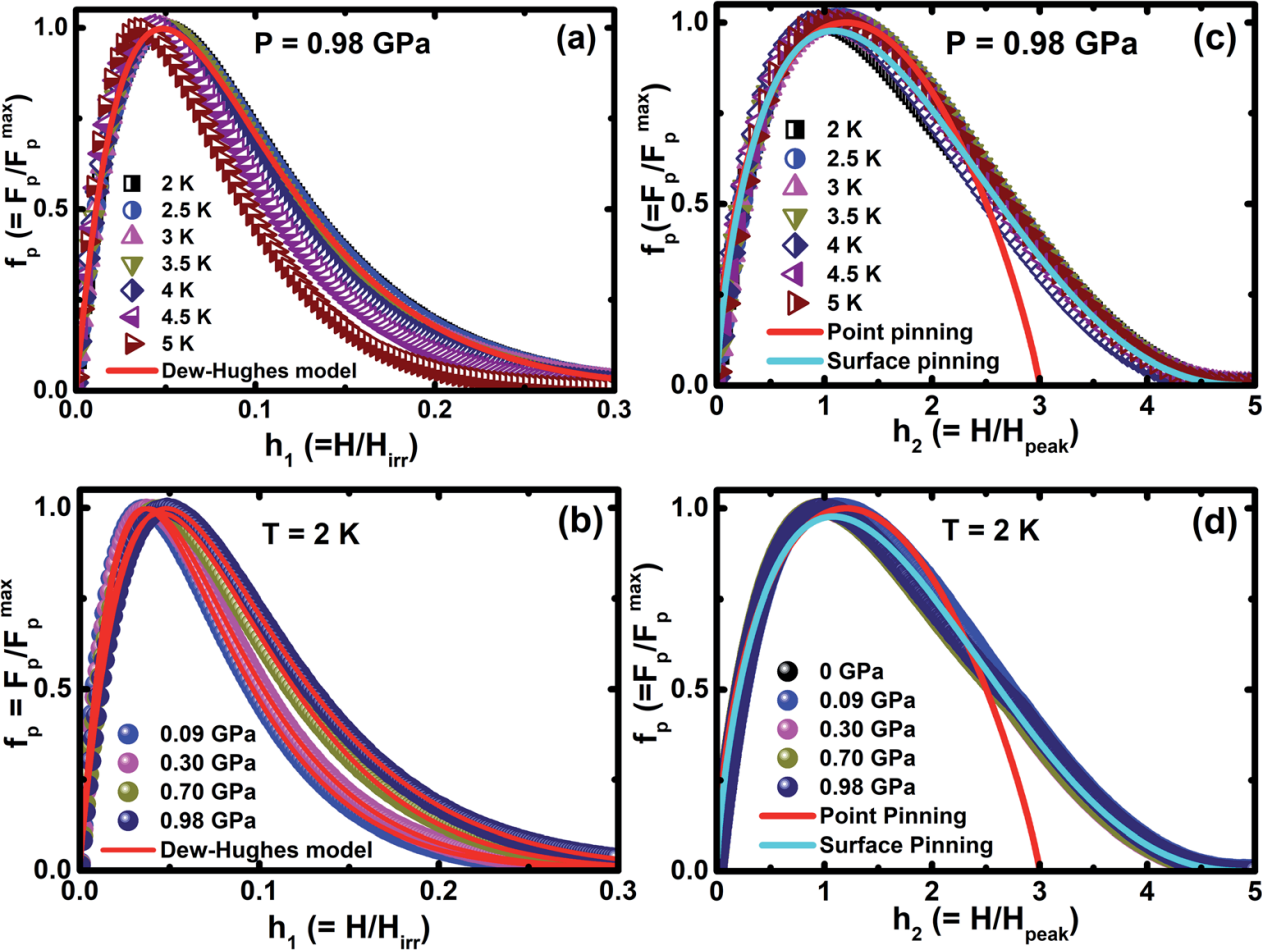
(a) Normalized pinning force *f*_p_ (*F*_p_/*F*^max^_p_) as a function of normalized magnetic field *h*_1_(*H*/*H*_irr_) for various temperatures at 0.98 GPa, (b) *f*_p_ = *F*_p_/*F*^max^_p_ dependence of *h*_1_ = *H*/*H*_irr_ at 2 K under various pressures up to ∼1 GPa and solid lines represent the scaling behavior for the pinning forces described by Dew-Hughes’ model, (c) normalized pinning force density *f*_p_ (*F*_p_/*F*^max^_p_) as a function of reduced field *h*_2_(*H*/*H*_peak_) for various temperatures at 0.98 GPa, (d) *f*_p_ = *F*_p_/*F*^max^_p_ dependence of *h*_2_ = *H*/*H*_peak_ at 2 K under various pressures up to ∼1 GPa and the solid lines represent the theoretical analysis of the method adapted by Higuchi *et al.* for Cr_*x*_NbSe_2_.

To understand the nature of pinning mechanism in more detail, it is useful to study the variation of vortex pinning force density (*F*_p_(*H*) = *J*_c_(*H*) × *μ*_0_*H*) as a function of magnetic field. The normalized magnetic field (*h* = *H*/*H*_irr_) dependence of normalized pinning force density (*f*_p_ = *F*_p_/*F*^max^_p_) at 0.98 GPa for various temperatures is shown in Fig. 8(a) and Fig. 8(b), which shows *f*_p_*vs. h*_1_ under various *P* up to 0.98 GPa at 2 K. We analyzed the pinning mechanism using Dew-Hughes model^67^*f*_p_(*h*) = *Ah*^*m*^(1 − *h*)^*n*^ + *Bh*^*p*^(1 − *h*)^*q*^ to fit our experimental data in order to describe the nature of pinning mechanism for unconventional superconductors. The exponents are characteristic for the dominant pinning mechanism, where *m* = 1, *n* = 2 and *p* = 0.5, *q* = 2 correspond to the normal point and surface pinning mechanism, respectively. By the application of external *P*, the pinning mechanism may be changed easily and it can be verified using the Dew-Hughes model; these results suggest that the occurrence of both point and surface pinning is observed. The *δl* pinning is more dominant because of the increase in core point pinning with the application of *P*. The normal core point pining is more prominent than the surface pinning mechanism on the application of *P*. These outcomes imply that Cr_*x*_NbSe_2_ have a number of deficient pinning centers in the high field region. It is outstanding that the external *P* influences the pinning centers, which in turn prompts the enhancement in *J*_c_. The best fit of the experimental data is achieved in the exponent range of 0.98 ≤ *m* ≤ 1.52 and 2.82 ≤ *n* ≤ 3.52: 0.48 ≤ *p* ≤ 0.31 and 1.69 ≤ *q* ≤ 2.03 in the pressure range of 0.09 GPa to 0.98 GPa. It reveals that the best fit exponents are close to the value predicted for the surface and point pinning and prove the coexistence of both pinning at high *P*. Further, for understanding the actual pinning mechanism of Cr_*x*_NbSe_2_ under *P*, we analyzed the peak positions at which the maximum pinning force is exhibited from the relation defined as *h*_max_ (*h*_max_*= p*/(*p* + *q*)). The values of *h*_max_ are 0.26 (0.09 GPa) and 0.30 (0.98 GPa) for point pinning, and 0.22 (0.09 GPa) and 0.13 (0.98 GPa) for surface pinning respectively. This confirms that core point pinning is actually the dominant pinning mechanism prevailing in the sample, which is caused by external *P*. We also have analyzed the pinning mechanism by using the method adopted by Higuchi *et al.*^68^ using the relations *f*_p_(*h*_2_) = (9/4)*h*_2_(1 − (*h*_2_/3))^2^ and *f*_p_(*h*_2_) = (25/16)*h*^0.5^_2_(1 − (*h*_2_/5))^2^ for point and surface pinning, respectively. Fig. 8(c) and (d) illustrates that both point and surface pinning coexist in ambient and high pressures, and it shows good agreement with the point pinning mechanism for various temperatures in the low field region with ambient and high pressures. The normalized magnetic field is higher than *H*_peak_; point pinning occurs in lower magnetic field and surface pinning is observed in higher magnetic field for all applied *P*. However, the samples do not exhibit strong pinning centres with the application of high field. These results suggest that both Dew-Hughes and Higuchi methods give similar nature of the flux pinning mechanism.

**Fig. 9:**
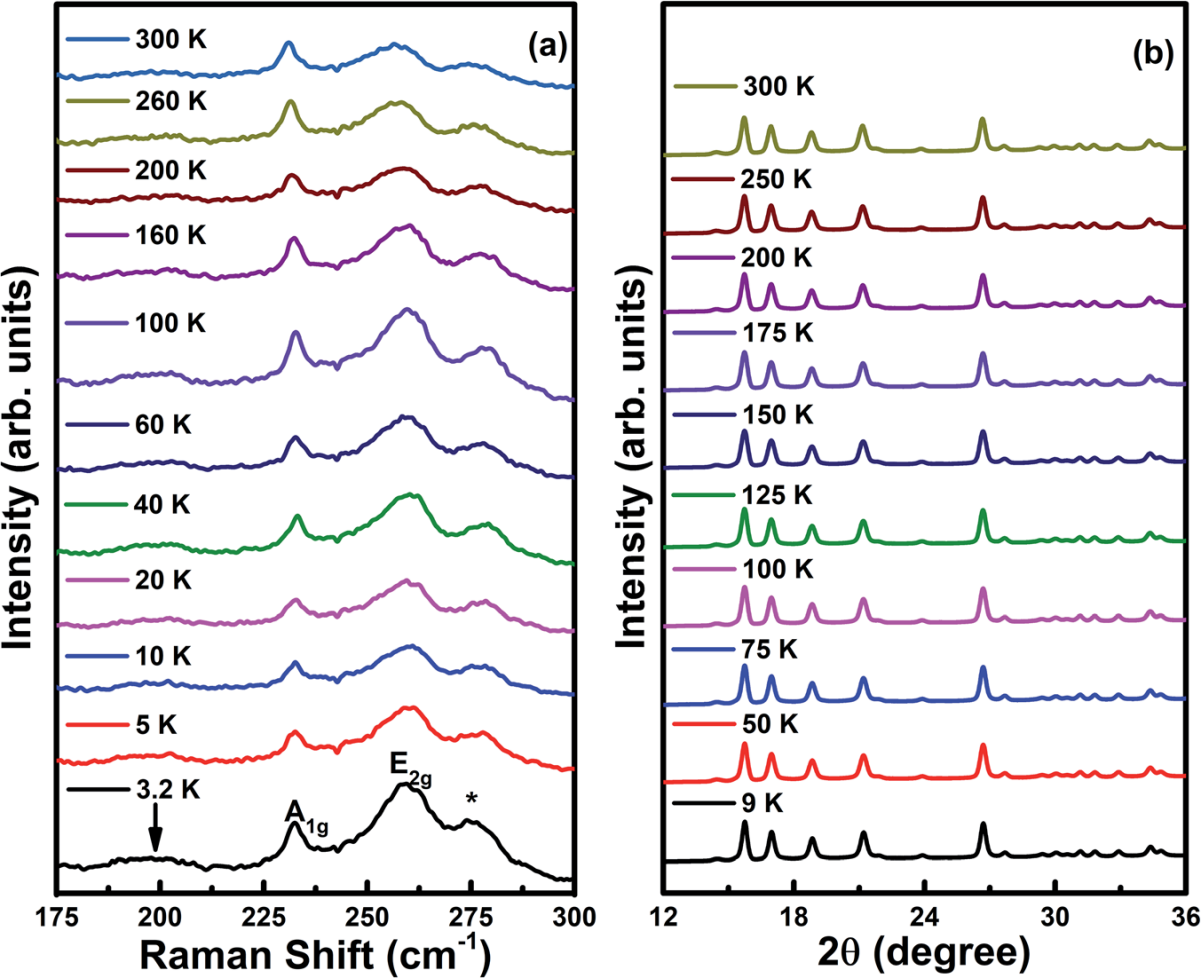
(a) Raman spectra (3.2 to 300 K) and (b) synchrotron powder X-ray diffraction pattern (9 to 300 K) at ambient pressure for Cr_*x*_NbSe_2_ single crystals.

The Raman spectra of Cr_*x*_NbSe_2_ single crystals are shown in Fig. 9(a) in the temperature range between 3.2 to 300 K. The prominent features observed below 300 cm^−1^ include two high energy in-plane (*E*_2g_) and out-of-plane (*A*_1g_) phonon modes at ∼256 cm^−1^ and ∼233 cm^−1^, respectively.^29,69^ The wide feature occurs at ∼190 cm^−1^, which is assigned as the soft mode and it is marked by a down arrow, which involves a second-order scattering process on two phonons of frequency *ω*_0_ at wavevector ∼(2/3)ΓM. These phonons consist of both intralayer and interlayer vibrations in the bulk NbSe_2_.^15,29,70^ The broad feature of the low energy mode is observed only in the parallel-polarization scattering geometry. The localized longitudinal-optical (LO) mode of Cr–Se appears in the Cr intercalated samples and it is marked as a star symbol at the Raman shift of 277 cm^−1^.^71^ The low temperature powder X-ray diffraction pattern for Cr_*x*_NbSe_2_ under various temperatures from 9 to 300 K is shown in Fig. 9(b) using the synchrotron source with wavelength 0.7962 Å and there are no impurity peaks found in Cr_*x*_NbSe_2_. These results confirm that there is no structural transition observed at room temperature and further decreasing the temperature down to 9 K does not show any changes in the XRD pattern. Hence, it confirms that there is no structural transition that occurs in this sample, as shown in Fig. 9(b).

**Fig. 10:**
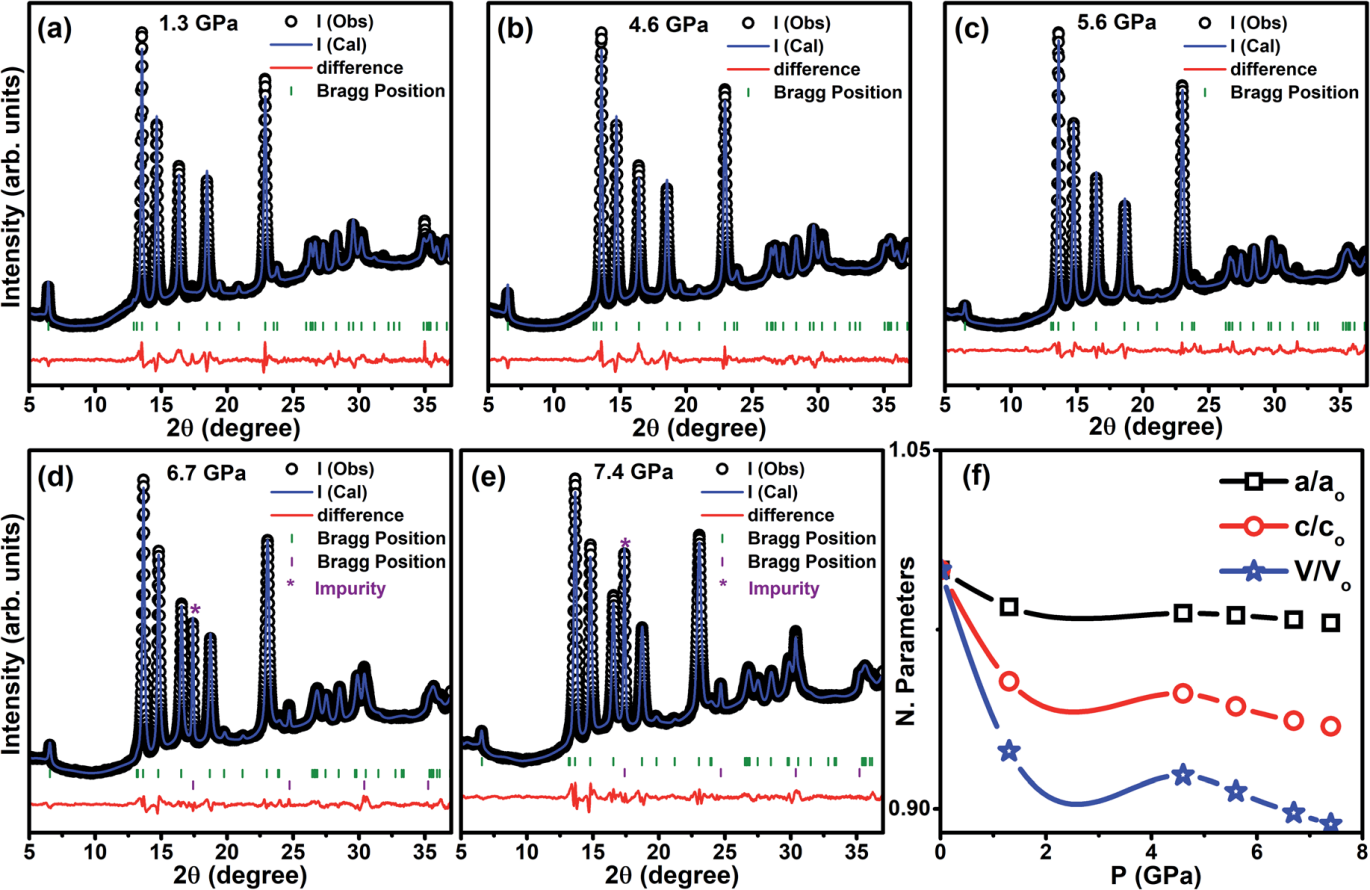
(a)–(e) Synchrotron powder ADXRD pattern for powder Cr_0.0009_NbSe_2_ at the selected pressures of 1.3 GPa, 4.6 GPa, 5.6 GPa, 6.7 GPa, and 7.4 GPa at 300 K and (f) the normalized lattice parameters (*a*/*a*_o_ and *c*/*c*_o_) and unit cell volume (*V*/*V*_o_) *versus* pressure. The parameters are normalized with the values obtained at ambient *P* and 300 K.

Fig. 10(a)–(f) shows the synchrotron powder ADXRD patterns for the Cr_0.0009_NbSe_2_ sample under various *P* up to ∼7.5 GPa; the two-dimensional (2D) image plate patterns are converted into one-dimensional patterns and plotted as a function of intensity using the Fit2D software package. As the pressure gradually increases, the XRD peaks shift steadily towards a higher angle. Apart from the peak shift, the diffraction peaks do not undergo any significant variations in the intensity up to the pressure of 7.4 GPa. All the XRD peaks of Cr_*x*_NbSe_2_ under various pressures at 300 K have been fitted by the Rietveld refinement method using FullProf software.^46^ The spectra are well fitted using a hexagonal crystalline structure with space group *P*6_3_/*mmc* with very good values of goodness of fit parameters. The lattice parameters are kept free during the fitting and it has been observed that the difference between the values of *a* and *c* parameters decreases with the application of pressure. The patterns obtained from both ambient and high-pressure regions reveal the absence of peak splitting, indicates the hexagonal phase, and it is found to be stable up to ∼7.4 GPa. All the XRD peaks and crystalline phases are well-matched with that shown by NbSe_2_ except for the 17.5° peak. In particular, the peak at 17.5° does not match with the NbSe_2_ pattern and it is due to the tungsten gasket used in the DAC. The variation in the normalized cell parameters and unit cell volume are plotted as a function of pressure, as shown in Fig. 10(e), and the estimated values are listed in Table 2. The *a*- and *b*-axis are less compressed under external *P* than the *c*-axis, which are made of the Nb–Se layer that is connected with a covalent bond. However, the Nb–Se layers are held together by van der Waals force parallel to the *c* axis and it leads to large and less compression along the *c* and *a*-axis, respectively. Transition metals have low electronegativity, which indicates that electrons can be transferred to the non-metallic constituent more easily, hence increasing the coulombic repulsion and causing difference in the compression among the stacking layers. Hence, a high anisotropy ratio is observed in the compressibility of Cr_*x*_NbSe_2_ layered superconducting materials.

**Table 2:** Unit cell, fitting parameters, and dislocation density obtained from the XRD pattern under ambient and high-pressure conditions

*P* (GPa)	*a* (Å)	*c* (Å)	*V* (Å^3^)	*χ* ^2^	*δ* (10^−6^ Å^−2^)
0	3.44325	12.54673	128.825	3.493	9.63
1.3	3.38991	11.9612	119.037	4.688	20.58
4.6	3.38072	11.89676	117.754	4.448	22.42
5.6	3.37769	11.82949	116.879	3.564	24.45
6.7	3.37163	11.75346	115.712	2.99	31.53
7.4	3.36697	11.72469	115.109	6.286	40.61

## Conclusions

We extensively studied the superconducting parameters such as *T*_c_, *H*_c2_, *H*_irr_, *J*_c_, and *F*_p_ under various hydrostatic pressures for the Cr_0.0009_NbSe_2_ single crystal. However, the relative contributions of the pinning mechanisms are strongly *P* dependent and it increases the *J*_c_ due to enhancement in both pinning centers and pinning strength. We found that the mechanism for both *δT*_c_ and *δl* pinning is associated with spatial fluctuations of mean free path and spatial fluctuations of the transition temperature from *J*_c_(*H*) at ambient and high *P* using collective pinning theory. The analysis was done using Dew-Hughes and Higuchi model, and it was proposed that the point and surface pinning coexist at ambient and high *P*. Further, point pinning is more dominant than surface pinning with the application of *P*. Hence, these results are important for practical application because they demonstrate that *P* can tune the formation of pinning centers. The investigation of structural analysis under high pressure up to 7.5 GPa demonstrated that the superconducting sample remains in the hexagonal crystal structure without any secondary phase and it shows high anisotropy along the *c*-axis than along the *ab*-plane.

## Conflicts of interest

There are no conflicts to declare.

## Supplementary Material

## References

[cit1] Ghiringhelli G., Le Tacon M., Minola M., Blanco-Canosa S., Mazzoli C., Brookes N. B., De Luca G. M., Frano A., Hawthorn D. G., He F., Loew T., Sala M. M., Peets D. C., Salluzzo M., Schierle E., Sutarto R., Sawatzky G. A., Weschke E., Keimer B., Braicovich L. (2012). Science.

[cit2] Le Tacon M., Bosak A., Souliou S. M., Dellea G., Loew T., Heid R., Bohnen K.-P., Ghiringhelli G., Krisch M., Keimer B. (2014). Nat. Phys..

[cit3] Arumugam S., Ganguli C., Thiyagarajan R., Bhoi D., Selvan G. K., Manikandan K., Pariari A., Mandal P., Uwatoko Y. (2017). Sci. Rep..

[cit4] Kalai Selvan G., Kanagaraj M., Esakki Muthu S., Jha R., Awana V. P. S., Arumugam S. (2013). Phys. Status Solidi RRL.

[cit5] Mizuguchi Y., Fujihisa H., Gotoh Y., Suzuki K., Usui H., Kuroki K., Demura S., Takano Y., Izawa H., Miura O. (2012). Phys. Rev. B: Condens. Matter Mater. Phys..

[cit6] Manikandan K., Shruti, Neha P., Kalai Selvan G., Wang B., Uwatoko Y., Ishigaki K., Jha R., Awana V. P. S., Arumugam S., Patnaik S. (2017). Europhys. Lett..

[cit7] Valla T., Fedorov A. V., Johnson P. D., Glans P. A., McGuinness C., Smith K. E., Andrei E. Y., Berger H. (2004). Phys. Rev. Lett..

[cit8] Manikandan K., Pervin R., Ganesan K. S., Murugesan K., Lingannan G., Verma A. K., Shirage P. M., Sonachalam A. (2018). Sci. Rep..

[cit9] Kim H. J., Kang W. N., Choi E. M., Kim M. S., Kim K. H. P., Lee S. I. (2001). Phys. Rev. Lett..

[cit10] Rotter M., Tegel M., Johrendt D. (2008). Phys. Rev. Lett..

[cit11] Kalai Selvan G., Thakur G. S., Manikandan K., Uwatoko Y., Haque Z., Gupta L. C., Ganguli A. K., Arumugam S. (2015). J. Phys. Soc. Jpn..

[cit12] Luo H., Strychalska-Nowak J., Li J., Tao J., Tomasz K., Cava R. J. (2017). Chem. Mater..

[cit13] Pervin R., Manikanadan K., Rana A. K., Kannan M., Arumugam S., Shirage P. M. (2017). Phys. Chem. Chem. Phys..

[cit14] Frindt R. F. (1972). Phys. Rev. Lett..

[cit15] Xi X., Zhao L., Wang Z., Berger H., Forró L., Shan J., Mak K. F. (2015). Nat. Nanotechnol..

[cit16] Weber F., Rosenkranz S., Castellan J. P., Osborn R., Hott R., Heid R., Bohnen K. P., Egami T., Said A. H., Reznik D. (2011). Phys. Rev. Lett..

[cit17] Arguello C. J., Rosenthal E. P., Andrade E. F., Jin W., Yeh P. C., Zaki N., Jia S., Cava R. J., Fernandes R. M., Millis A. J., Valla T., Osgood R. M., Pasupathy A. N. (2015). Phys. Rev. Lett..

[cit18] Littlewood P. B., Varma C. M. (1982). Phys. Rev. B: Condens. Matter Mater. Phys..

[cit19] Grasset R., Cea T., Gallais Y., Cazayous M., Sacuto A., Cario L., Benfatto L., Méasson M.-A. (2018). Phys. Rev. B.

[cit20] Mattheiss L. F. (1973). Phys. Rev. B: Solid State.

[cit21] Mattheiss L. F. (1973). Phys. Rev. Lett..

[cit22] Den Berg J. M. V.-V. (1972). J. Less-Common Met..

[cit23] Sugawara K., Yokota K., Tanokura Y., Sekine T. (1993). J. Low Temp. Phys..

[cit24] Yan D., Lin Y., Wang G., Zhu Z., Wang S., Shi L., He Y., Li M.-R., Zheng H., Ma J., Jia J., Wang Y., Luo H. (2019). Supercond. Sci. Technol..

[cit25] Galvan D. H., Li S., Yuhasz W. M., Kim J. H., Maple M. B., Adem E. (2003). Phys. C.

[cit26] Cho K., Kończykowski M., Teknowijoyo S., Tanatar M. A., Guss J., Gartin P. B., Wilde J. M., Kreyssig A., McQueeney R. J., Goldman A. I., Mishra V., Hirschfeld P. J., Prozorov R. (2018). Nat. Commun..

[cit27] Pervin R., Manikanadan K., Rana A. K., Arumugam S., Shirage P. M. (2018). Mater. Res. Express.

[cit28] Ganguli S. C., Singh H., Ganguly R., Bagwe V., Thamizhavel A., Raychaudhuri P. (2016). J. Phys.: Condens. Matter.

[cit29] Naik S., Pradhan G. K., Bhat S. G., Behera B. C., Kumar P. S. A., Samal S. L., Samal D. (2019). Phys. C.

[cit30] Iavarone M., Di Capua R., Karapetrov G., Koshelev A. E., Rosenmann D., Claus H., Malliakas C. D., Kanatzidis M. G., Nishizaki T., Kobayashi N. (2008). Phys. Rev. B: Condens. Matter Mater. Phys..

[cit31] Arumugam S., Manikandan K., Ishigaki K., Gouchi J., Pervin R., Selvan G. K., Shirage P. M., Uwatoko Y. (2019). Sci. Rep..

[cit32] Chu C. W., Diatschenko V., Huang C. Y., DiSalvo F. J. (1977). Phys. Rev. B: Solid State.

[cit33] Arumugam S., Môri N., Takeshita N., Takashima H., Noda T., Eisaki H., Uchida S. (2002). Phys. Rev. Lett..

[cit34] Gao L., Xue Y. Y., Chen F., Xiong Q., Meng R. L., Ramirez D., Chu C. W., Eggert J. H., Mao H. K. (1994). Phys. Rev. B: Condens. Matter Mater. Phys..

[cit35] Monteverde M., Acha C., Núñez-Regueiro M., Pavlov D. A., Lokshin K. A., Putilin S. N., V Antipov E. (2005). Europhys. Lett..

[cit36] Drozdov A. P., Eremets M. I., Troyan I. A., Ksenofontov V., Shylin S. I. (2015). Nature.

[cit37] Miyoshi K., Morishita K., Mutou E., Kondo M., Seida O., Fujiwara K., Takeuchi J., Nishigori S. (2014). J. Phys. Soc. Jpn..

[cit38] Gooch M., Lv B., Lorenz B., Guloy A. M., Chu C.-W. (2008). Phys. Rev. B: Condens. Matter Mater. Phys..

[cit39] Takahashi H., Okada H., Igawa K., Arii K., Kamihara Y., Matsuishi S., Hirano M., Hosono H., Matsubayashi K., Uwatoko Y. (2008). J. Phys. Soc. Jpn..

[cit40] Garbarino G., Toulemonde P., Álvarez-Murga M., Sow A., Mezouar M., Núñez-Regueiro M. (2008). Phys. Rev. B: Condens. Matter Mater. Phys..

[cit41] Tissen V. G., Osorio M. R., Brison J. P., Nemes N. M., García-Hernández M., Cario L., Rodière P., Vieira S., Suderow H. (2013). Phys. Rev. B: Condens. Matter Mater. Phys..

[cit42] Jung S. G., Kang J. H., Park E., Lee S., Lin J. Y., Chareev D. A., Vasiliev A. N., Park T. (2015). Sci. Rep..

[cit43] Freitas D. C., Rodière P., Osorio M. R., Navarro-Moratalla E., Nemes N. M., Tissen V. G., Cario L., Coronado E., García-Hernández M., Vieira S., Núñez-Regueiro M., Suderow H. (2016). Phys. Rev. B.

[cit44] Yamaya K. (1974). J. Phys. Soc. Jpn..

[cit45] Abdel-Hafiez M., Zhao X.-M., Kordyuk A. A., Fang Y.-W., Pan B., He Z., Duan C.-G., Zhao J., Chen X.-J. (2016). Sci. Rep..

[cit46] Rodríguez-Carvajal J. (1993). Phys. B.

[cit47] Shirley D. A. (1972). Phys. Rev. B: Solid State.

[cit48] Buabthong P., Stasiewicz N. B., Mitrovic S., Lewis N. S., Buabthong P., Stasiewicz N. B., Mitrovic S. (2017). Surf. Sci. Spectra.

[cit49] Hüfner S. , Photoelectron Spectroscopy: Principles and Applications, Springer, Berlin, Heidelberg, 2003

[cit50] Kulkarni S. K., Thube M. G., Nigavekar A. S. (1988). Phys. Rev. B: Condens. Matter Mater. Phys..

[cit51] Shruti, Maurya V. K., Neha P., Srivastava P., Patnaik S. (2015). Phys. Rev. B: Condens. Matter Mater. Phys..

[cit52] Ma J. Z., Van Roekeghem A., Richard P., Liu Z. H., Miao H., Zeng L. K., Xu N., Shi M., Cao C., He J. B., Chen G. F., Sun Y. L., Cao G. H., Wang S. C., Biermann S., Qian T., Ding H. (2014). Phys. Rev. Lett..

[cit53] Burrard-Lucas M., Free D. G., Sedlmaier S. J., Wright J. D., Cassidy S. J., Hara Y., Corkett A. J., Lancaster T., Baker P. J., Blundell S. J., Clarke S. J. (2013). Nat. Mater..

[cit54] Zhang A., Xia T., Liu K., Tong W., Yang Z., Zhang Q. (2013). Sci. Rep..

[cit55] Ying T., Chen X., Wang G., Jin S., Lai X., Zhou T., Zhang H., Shen S., Wang W. (2013). J. Am. Chem. Soc..

[cit56] Werthamer N. R., Helfand E., Hohenberg P. C. (1966). Phys. Rev..

[cit57] Gruenberg L. W., Gunther L. (1966). Phys. Rev. Lett..

[cit58] Yeshurun Y., Bontemps N., Burlachkov L., Kapitulnik A. (1994). Phys. Rev. B: Condens. Matter Mater. Phys..

[cit59] Sundar S., Salem-Sugui S., Amorim H. S., Wen H.-H., Yates K. A., Cohen L. F., Ghivelder L. (2017). Phys. Rev. B.

[cit60] Tinkham M. , Introduction to Superconductivity: Second Edition, Dover Books on Physics, Dover Publications, New York, 2nd edn, 2004

[cit61] Brandt E. H. (1999). Phys. Rev. B: Condens. Matter Mater. Phys..

[cit62] Hu C.-R. (1972). Phys. Rev. B: Solid State.

[cit63] Bean C. P. (1964). Rev. Mod. Phys..

[cit64] Blatter G., Feigel’man M. V., Geshkenbein V. B., Larkin A. I., Vinokur V. M. (1994). Rev. Mod. Phys..

[cit65] Kramer E. J. (1973). J. Appl. Phys..

[cit66] Pan V., Cherpak Y., Komashko V., Pozigun S., Tretiatchenko C., Semenov A., Pashitskii E., Pan A. V. (2006). Phys. Rev. B: Condens. Matter Mater. Phys..

[cit67] Dew-Hughes D. (1974). Philos. Mag..

[cit68] Higuchi T., Yoo S. I., Murakami M. (1999). Phys. Rev. B: Condens. Matter Mater. Phys..

[cit69] Wu Y., An M., Xiong R., Shi J., Zhang Q. M. (2008). J. Phys. D: Appl. Phys..

[cit70] Tsang J. C., Smith J. E., Shafer M. W. (1976). Phys. Rev. Lett..

[cit71] Rai B. K., Katiyar R. S., Chen K. T., Burger A. (1998). J. Appl. Phys..

